# Synthesis, Reactions and Biological Evaluation of Some New Naphtho[2,1-*b*]furan Derivatives Bearing a Pyrazole Nucleus

**DOI:** 10.3390/molecules16010307

**Published:** 2011-01-05

**Authors:** Ashraf H. F. Abd El-Wahab, Zarrag Isa A. Al-Fifi, Ahmed H. Bedair, Fawzy M. Ali, Ahmed H. A. Halawa, Ahemed M. El-Agrody

**Affiliations:** 1 Department of Chemistry, Faculty of Science, Jazan University, Jazan 2097, Saudi Arabia; 2 Department of Biology, Faculty of Science, Jazan University, Jazan 2097, Saudi Arabia; 3 Department of Chemistry, Faculty of Science, Al-Azhar University, Nasr City 11884, Cairo, Egypt

**Keywords:** Vilsmeier reaction, 2-[3-(naphtho[2,1-*b*]furan-2-yl)-1-phenyl-1*H*-pyrazol-4-yl)methylene]malononitrile, 1,3,4-thiadiazol-2-ylacetamide, antimicrobial activity

## Abstract

Vilsmeier formylation of 2-(1-phenylhydrazonoethyl)naphtho[2,1-*b*]furan (**2**) gave 3-naphtho[2,1-*b*]furan-2-yl-1-phenyl-1*H*-pyrazole-4-carbaldehyde (**3**), which was reacted with C- and *N*-nucleophiles to afford naphthofuranpyrazol derivatives **4-8**. Treatment of 2-[(3-(naphtho[2,1-*b*]furan-2-yl)-1-phenyl-*1H-*pyrazol-4-yl)methylene]-malononitrile (**4a**) with reactants having active hydrogen and Et_3_N gave the corresponding pyrazoline, pyran and chromene addition product derivatives **10**, **12** and **13**, consisting of three different connected heterocyclic moieties. Reaction of 1-((3-(naphtho[2,1-b]furan-2-yl)-1-phenyl-1*H*-pyrazol-4-yl) methylene)-2-phenylhydrazone (**6b**) with AcONa and ethyl bromoacetate or chloroacetone afforded the thiazolidinone and methylthiazole derivatives **14** and **15**, respectively. In addition, intramolecular cyclization of **6d** with Ac_2_O afford the corresponding 1,3,4-thiadiazol-2-yl acetamide derivative **16**. The structures of the synthesized compounds were confirmed by IR, ^1^H-NMR/^13^C-NMR and mass spectral studies. Compound **14** showed promising effects against the tested Gram positive and negative bacteria and fungi.

## Introduction

Naphthofuran derivatives exhibit very potent antibacterial [1,2,3], genotoxic [4,5,6] and anticancer activity [7,8,9], but some were shown to be mutagenic in bacteria [10,11,12,13,14,15]. The present investigation deals with the synthesis of some new naphtho[2,1-*b*]furan derivatives bearing pyrazole nuclei attached to different heterocyclic moieties and their biological activities. 

## Results and Discussion

**Scheme 1:**
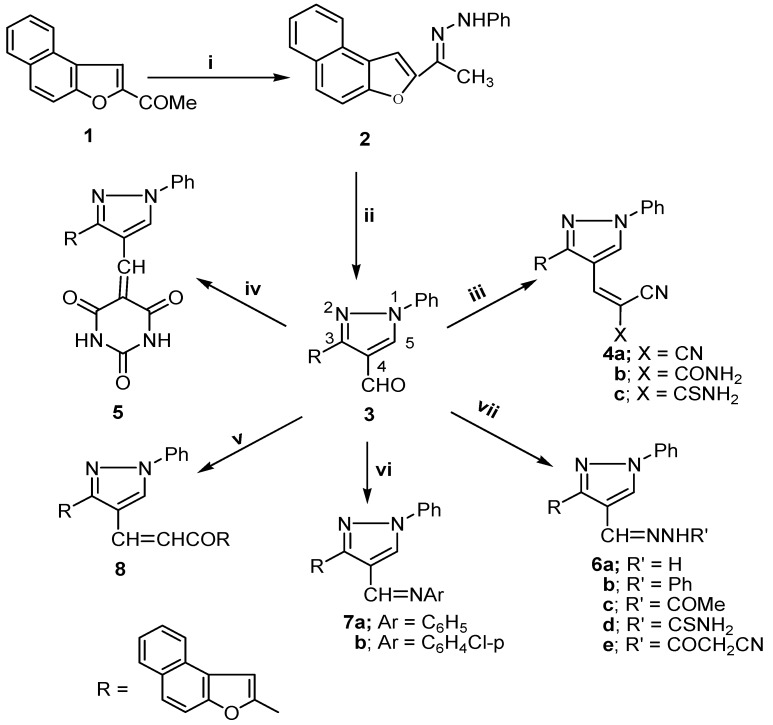
Synthesis of naphtho[2,1-*b*]furan pyrazole derivatives.

Condensation of 2-acetylnaphtho[2,1-*b*]furan (**1**) [16] with phenylhydrazine afforded 2-(1-phenylhydrazonoethyl)naphtho[2,1-*b*]furan (**2**). Vilsmeier formylation [17] of the latter afforded 3-(naphtho-[2,1-*b*]furan-2-yl)-1-phenyl-1*H*-pyrazole-4-carboxaldehyde (**3**). Condensation of **3** with a variety of C-nucleophiles, namely malononitrile, cyanoacetamide, cyanothioacetamide, barbituric acid and 2-acetylnaphtho[2,1-*b*]furan (**1**) give the condensation products **4**, **5** and **8**, while reactions with *N*-nucleophiles, namely hydrazine derivatives or amines, afforded the condensation products **6** and **7** (Scheme 1).

**Scheme 2:**
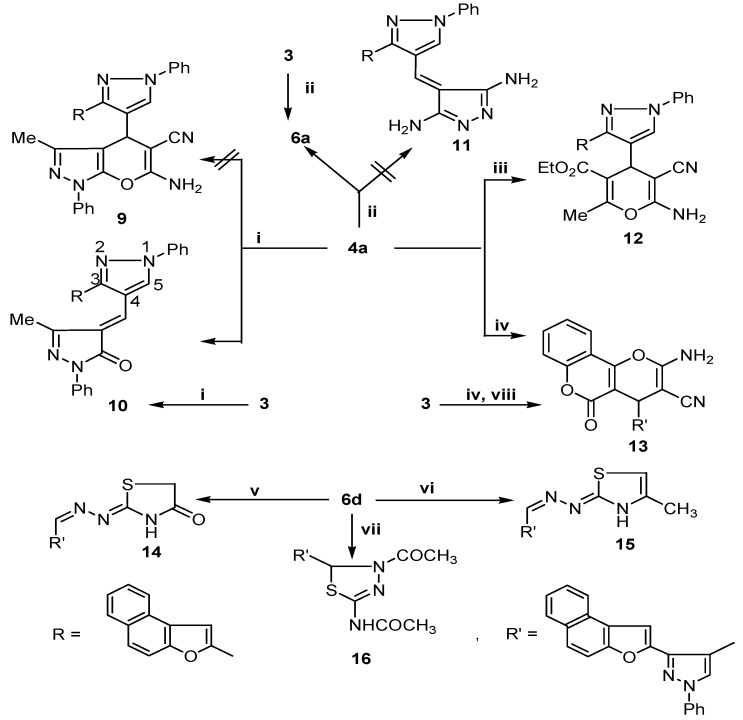
Synthesis of pyran, pyrazole and thiazole derivatives.

Compounds **4a** and **6d** were used as key intermediates in the synthesis of novel pyran, pyrazole and thiazole derivatives via their interaction with different reagents. Thus, the reaction of **4a** with 3-methyl-1-phenyl-2-pyrazolin-5-one in the presence of triethylamine did not give the expected pyrazolopyran **9** and instead, only one compound was isolated, identified as 4-[3-(naphtho[2,1-*b*]furan-2-yl)-1-phenyl-1*H*-pyrazol-4-yl]methylene-3-methyl-1-phenyl-2-pyrazoline-5-one (**10**), while with hydrazine hydrate in refluxing ethanol it furnished **6a** (as verified by m.p. and mixed m.p.) instead of the pyrazole derivative **11**. The formation of **6a** and **10** were assumed to proceed via elimination of malononitrile [18] and their structures were further confirmed by independent synthesis via direct condensation of **3** with hydrazine hydrate (Scheme 1) or with 3-methyl-1-phenyl-2-pyrazolin-5-one (Scheme 2), respectively The reaction of **4a** with ethyl acetoacetate in dry methylene chloride containing triethylamine gave the pyrazolopyran derivative **12**, while with 4-hydroxycoumarin under Michael reaction conditions it afforded the 4*H*-pyran derivative **13** (Scheme 2). The structure of **13** was further verified by m.p. and mixed m.p after independent synthesis via direct condensation of **3** with 4-hydroxycoumarin in the presence of malononitrile and a few drops of piperidine as a base (a one pot reaction).

When thiosemicarbazone derivative **6d** was allowed to react with ethyl bromoacetate or chloroacetone in the presence of fused sodium acetate, it gave the corresponding thiazolidinone and thiazoline derivatives **14** and **15**, respectively, while with refluxing acetic anhydride it afforded the thiadiazole derivative **16** (Scheme 2). These reactions were assumed to proceed via *S*-alkylation followed by intramolecular cyclization with concomitant loss of an alcohol molecule and/or dehydration.

**Scheme 3:**
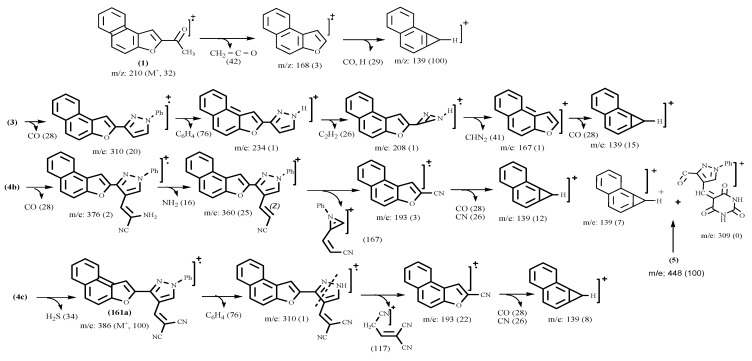

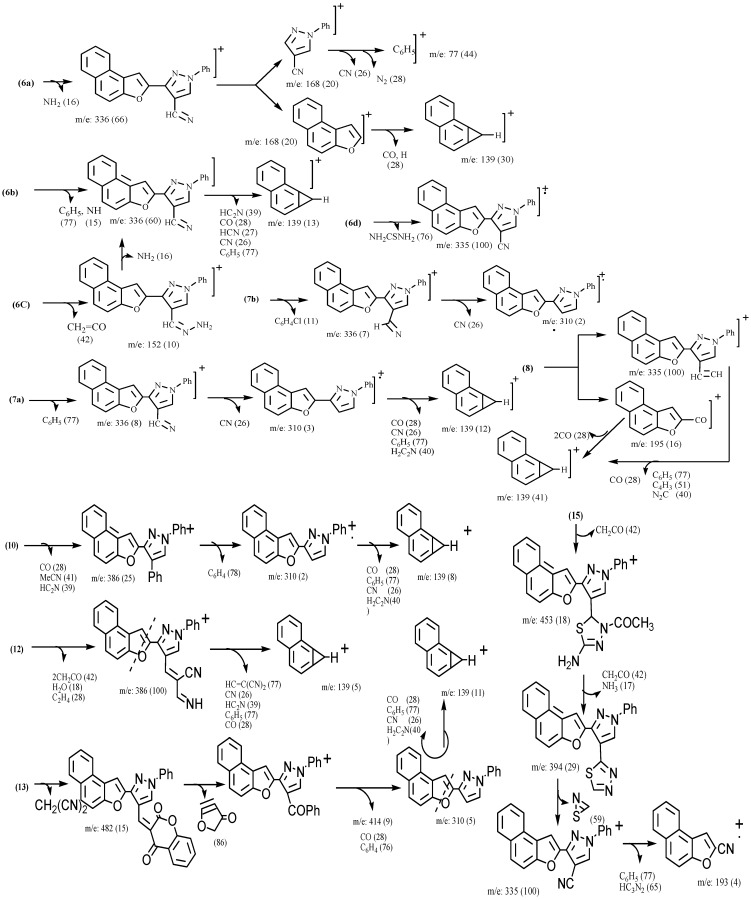
Proposed fragmentation pathways for the EI spectra of the substituted naphtho[2,1-*b*]furans **3-16**.

Structures **2, 3, 4, 5, 6, 7** and **8** were established by spectral and physical data (Table I and Table II). The mass spectra of **3, 4b,c, 5, 6a-d, 7a,b** and **8** showed the corresponding molecular ion peaks at *m/z* 338 (M^+^, 100%), *m/z* 420 (M^+^, 35 %), *m/z* 404 (M^+^, 100 %), *m/z* 448 (M^+^, 100%), *m/z* 352 (M^+^, 100 %), *m/z* 404 (M^+^, 100 %), *m/z* 394 (M^+^, 100%), *m/z* 411 (M^+^, 2 %), *m/z* 413 (M^+^, 100 %), *m/z* 447(M^+^, 100%) and *m/z* 530 (M^+^, 65 %), respectively. The fragmentation patterns of compounds **3, 4b,c, 5, 6a-d, 7a,b** and **8** are illustrated in Scheme 3.

Structures **10**, **11**, **12**, **13**, and **16** were established by spectral data (Table I and Table II). The mass spectra of **10**, **12**, **13** and **15** showed molecular ion peaks at *m/z*; 494 (M^+^, 100), *m/z*; 516 (M^+^, 2), *m/z*; 548 (M^+^, 2), and *m/z*; 495 (M^+^, 45), respectively. The fragmentation pattern of compounds **10**, **12**, **13** and **15** are illustrated in Scheme 3.

### Biological activities

Compound **14** which contains the thiazolidin-4-one nucleus showed highest antibacterial activity (+++ ve inhibition zone was between 12–15 mm) against *Bacillus subtilis, Staphylococcus aureus, Escherichia coli, Pseudomonas aeuroginosa, Candida albicans and Aspergillus niger.* Compound **2** showed moderate inhibition (++ ve inhibition zone up to 8 mm) against *Bacillus subtilis, Staphylococcus aureus, Escherichia coli and Pseudomonas aeuroginosa* , the remaining tested compounds **4a-c, 5, 6, 7a,b, 8a-c, 10, 12, 13** showed no activities against any of the test microorganisms. 

## Experimental

### General

Melting points were measured on a Stuart Scientific Co (UK) melting point apparatus and are uncorrected. The IR spectra were recorded on a Shimadzu IR 440, spectrophotometer (Shimadzu, Japan) in KBr. The ^1^H-NMR/^13^C-NMR spectra were measured on a Varian Mercury (300 MHz) spectrometer (Varian, UK), using TMS as an internal standard and DMSO-d_6_ as solvent. Mass spectra were run on a Shimadzu GC-Ms QP 1000 EX mass spectrometer. Microanalytical data were obtained from the Microanalytical Unit Center, Faculty of Science, Cairo University (Egypt). Spectral and microanalytical data are given in Table I, Table II and Table III. The paper discs were manufactured by Bristol-Myers Squibb, Giza, Egypt.

*Synthesis of 2-acetylnaphtho[2,1-b]furan* (**1**): A mixture of 2-hydroxy-1-naphthaldehyde (0.01 mol), chloroacetone (0.01 mol) and anhydrous potassium carbonate (0.02 mol) in anhydrous acetone (50 mL) was refluxed for 8 hours. The mixture was allowed to cool and poured onto crushed ice (50 gm) and water (100 mL) then acidified with conc. HCl and the solid product was formed was filtered off, washed with water and recrystallized from ethanol to give **1** (90% yield); IR: 1,666 (CO): ^1^H-NMR: 8.59–7.70 (m, 6H, Ar-H), 7.89 (s, 1H, H-3), 2.62 (s, 3H, Me): ^13^C-NMR: 187.19 (CO), 153.36 (C-9a), 151.90 (C-2), 130.11 (C-3a), 129.90 (C-8), 128.98 (C-7), 127.85 (C-7a), 127.52 (C-6), 125.60 (C-5), 123.64 (C-4), 122.61 (C-3b), 113.69 (C-3), 112.74 (C-9), 26.33 (Me). 

*Synthesis of 2-(1-phenylhydrazonoethyl)naphtho[2,1-b]furan* (**2**): A mixture of **1** (1.72 g, 0.01 mol) and phenylhydrazine (1.08 g, 0.01 mol) in ethanol (50 mL) was refluxed for 2 h, the solid that separated on heating was filtered off and recrystallized from EtOH to give **2** (80% yield); IR : 3,459, 3,346 (NH),1,601 (C=N); ^1^H-NMR: 8.17–6.92 (m, 12H, Ar-H+NH), 7.70 (s, 1H, H-3), 2.35 (s, 3H, Me); ^13^C-NMR: 154.81 (C-9a), 151.72 (C=N), 145.45 (C-2), 132.94 (C-1`), 130.01 (C-3a), 128.94 (C-8), 128.66 (C-3`,5`), 127.06 (C-7a), 126.44 (C-7), 125.28 (C-6), 124.75 (C-5), 123.97 (C-3b), 123.65 (C-4), 119.39 (C-4`), 113.04 (C-3), 112.22 (C-9), 102.89 (C-2`,6`), 12.80 (Me).

*Synthesis of 3-naphtho[2,1-b]furan-2-yl-1-phenyl-1H-pyrazole-4-carbaldehyde* (**3**): To ice cold dimethylformamide (0.01 mol) was added dropwise with stirring phosphorus oxychloride (0.01 mol) over a period of 30 min. stirring was continued for further 45 min keeping the reaction mixture at 0 °C. The hydrazone **2** (3 g, 0.01 mol) was then added and the reaction mixture was allowed to attain room temperature. The mixture was heated at 90 °C for 2 h, allowed to cool and poured onto crushed ice (50 gm) and water (100 mL), the solid product that was formed was filtered off and washed with water and recrystallized from EtOH to give **3** (70% yield); IR: 1,670 (CO): ^1^H-NMR: 10.33 (s, 1H, CHO), 9.37 (s, 1H, H-5), 8.43–7.47 (m, 11H, Ar-H), 7.92 (s, 1H, H-3`). 

*Synthesis of 2-((3-(naphtho[2,1-b]furan-2-yl)-1-phenyl-1H-pyrazol-4-yl)methylene) malononitrile* (**4a**), *2-cyano-3-(3-(naphtho[2,1-b]furan-2-yl)-1-phenyl-1H-pyrazol-4-yl) acrylamide* (**4b**) *and 2-cyano-3-(3-(naphtho[2,1-b]furan-2-yl)-1-phenyl-1H-pyrazol-4-yl) prop-2-enethioamide* (**4c**): A mixture of aldehyde **3** (3.38 g, 0.01 mol) and malononitrile, cyanoacetamide or cyanothioacetamide (0.01 mol) in ethanol (30 mL) and few drops of piperidine was refluxed for 3 h, The resulting solid after cooling was collected and recrystallized from the proper solvent to give **4a-c** (70–80% yield);. **4a**: IR: 2,208, 2,200 (2 CN): ^1^H-NMR: 10.15 (s, 1H, CH=C), 8.65 (s, 1H, H-5), 8.55–7.52 (m, 12H, Ar-H). **4b**: IR: 3,464, 3,368 (NH_2_), 2,210 (CN), 1,698 (CO): ^1^H-NMR: 9.19 (s, 1H, CH=C), 8.60 (s, 1H, H-5), 8.55–7.52 (m, 14H, Ar-H +NH_2_). **4c**: IR: 3,344, 3,286 (NH_2_), 2,212 (CN), 1,340 (C=S): ^1^H-NMR: 10.12 (br, 2H, NH_2_), 9.67 (s, 1H, CH=C), 9.19 (s, 1H, H-5), 8.59–7.48 (m, 12H, Ar-H).

*Synthesis of 5-((3-(naphtho[2,1-b]furan-2-yl)-1-phenyl-1H-pyrazol-4-yl) methylene) pyrimidine-2,4,6(1H,3H,5H)trione* (**5**): A mixture of aldehyde **3** (3.38 g, 0.01 mol) and barbituric acid (0.01 mol) in ethanol (30 mL) containing triethylamine (0.01 mL) was heated under reflux for 1h. The solid separated on heating was filtered off and recrystallized from ethanol to give **5** (80% yield). IR: 3,200 (NH), 1,700 (CO), 1,670 (CO): ^1^H-NMR: 11.38, 11.30 (2s, 2H, 2NH), 9.67 (s, 1H, CH=C), 8.77 (s, 1H, H-5), 8.27–7.36 (m, 12H, Ar-H).

*Synthesis of 1-((3-(naphtho[2,1-b]furan-2-yl)-1-phenyl-1H-pyrazol-4-yl)methylene) hydrazone* (**6a**), *1-((3-(naphtho[2,1-b]furan-2-yl)-1-phenyl-1H-pyrazol-4-yl) methylene)-2-phenylhydrazone* (**6b**), *N’-((3-(naphtho[2,1-b]furan-2-yl)-1-phenyl-1H-pyrazol-4-yl)methylene) acetohydrazone* (**6c**), *1-((3-(naphtha[2,1-b]furan-2-yl)-1-phenyl-1H-pyrazol-4-yl)methylene) thiosemicarbazone* (**6d**) *and 2-cyano-N’-((3-(naphtho[2,1-b]furan-2-yl)-1-phenyl-1H-pyrazol-4-yl)methylene)acetohydrazone* (**6e**): A mixture of aldehyde **3** (3.38 g, 0.01 mol) and hydrazine hydrate, phenyl hydrazine, acetylhydrazine, thiosemicarbazide or cyanoacetic acid hydrazide (0.01 mol) in ethanol (30 mL) was refluxed for 3 h, the solid separated on heating was filtered off and recrystallized from a suitable solvent to give **6a-e** (75–80 %). **6a**: IR: 3,400, 3,364 (NH_2_), 1,598 (C=N),^1^H-NMR: 8.78 (s, 1H, CH=N), 8.41 (s, 1H, H-5), 8.37–7.33 (m, 12H, Ar-H), 6.86 (brs, 2H, NH_2_). **6b**: IR: 3,320 (NH), 1,602 (C=N); ^1^H-NMR: 10.39 (brs, 1H, NH), 9.01 (s, 1H, CH=N), 8.40 (s, 1H, H-5), 8.36-7.17 (m, 12H,Ar-H). **6c**: IR: 3,156 (NH), 1,686 (CO); ^1^H-NMR: 11.60 (brs, 1H, NH), 11.26 (s, 1H, CH=N), 9.17 (s, 1H, H-5), 9.06-7.34 (m, 12H, Ar-H), 2.26 (s, 3H, Me). **6d**: IR: 3,420, 3,374, 3,262, 3,178 (NH, NH_2_), 1,334 (C=S); ^1^H-NMR: 11.40 (s, 1H, NH), 9.27 (s, 1H, CH=N), 8.72 (s, 1H, H-5), 8.32 (brs, 2H, NH_2_), 8.41–7.41 (m, 12H, Ar-H). **6e**: IR: 3,310, 3,210, (NH), 2,256 (CN), 1,666 (CO); ^1^H-NMR: 12.02 (brs, 1H, NH), 9.10 (s, 1H, CH=N), 8.9 (s, 1H, CH-pyrazole), 8.93-7.41 (m, 12H, Ar-H), 4.28 (s, 2H, CH_2_).

*Synthesis of N-((3-(naphtho[2,1-b]furan-2-yl)-1-phenyl-1H-pyrazol-4-yl)methylene) aniline/4-chloro-aniline* (**7a,b**)**:** A mixture of aldehyde **3** (3.38 g, 0.01 mol) and the appropriate aromatic amine, namely aniline or *p*-chloroaniline (0.01 mol) in ethanol (40 mL) was refluxed for 1 h. The obtained product was collected and recrystallized from the proper solvent to give **7a,b**. (80–85% yield)) **7a**: IR: 1,614 (C=N); ^1^H-NMR: 9.40 (s, 1H, CH=N), 8.96 (s, 1H, H-5), 8.33–7.36 (m, 17H, Ar-H). **7b**: IR: 1,620 (C=N); ^1^H-NMR: 9.25 (s, 1H, CH=N), 8.95 (s, 1H, H-5), 8.36–7.35 (m, 16H, Ar-H).

*Synthesis of 1-(naphtho[2,1-b]furan-2-yl)-3-(3-(naphtho[2,1-b]furan-2-yl)-1-phenyl-1H-pyrazol-4-yl)prop-2-en-1-one* (**8**): A mixture of 2-acetyl naphtho[2,1-b]furan (**1**, 0.01 mol) with aldehyde **3** (0.01 mol) in ethanol (30 mL) and few drops of piperidine was heated under reflux for 1 h. The solid separated on heating was filtered off and recrystallized from dioxane to give **8** (80 % yield): IR: 1,662 (CO), ^1^H-NMR: 8.96 (s, 1H, H-5), 8.30–7.20 (m, 19H, Ar-H), 5.93–5.69(dd, 2H, CH=CH, *J* = 11.4 Hz).

*Synthesis of 4-[3-(naphtho[2,1-b]furan-2-yl)-1-phenyl-1H-pyrazol-4-yl]methylene-3-methyl-1-phenyl-2-pyrazoline-5-one* (**10**): To a mixture of **4a** (0.01mol) and 3-methyl-1-phenyl-2-pyrazolin-5-one (0.01 mol) in ethanol (30 mL), triethylamine (0.01 mL) was added. The resulting mixture was heated under reflux for 30 min and left to cool. The precipitated product was collected and recrystallized from acetic acid to give **10** (85 %). IR: 1,678 (CO), 1,610 (C=N); ^1^H-NMR: 10.09 (s, 1H, CH=C), 8.41 (s, 1H, H-5), 8.14–7.18 (m, 17H, Ar-H), 2.3 (s, 3H, CH_3_).

*Synthesis of ethyl 6-amino-5-cyano-2-methyl-4-(3-(naphtho[2,1-b]furan-2-yl)-1-phenyl-1H-pyrazol-4-yl)-4H-pyran-3-carboxylate* (**12**): To a solution of **4a** (0.01 mol) and ethyl acetoacetate (0.01 mol) in methylene chloride (50 mL), triethylamine (0.01 mL) was added. The mixture was heated under reflux for 4 h, concentrated and allowed to cool. The precipitated product was collected and recrystallized from ethanol to give **12** (70 % yield). IR: 3,402, 3,328, 3,206 (NH_2_), 2,194 (CN), 1,690 (CO); ^1^H-NMR: 8.58 (s, 1H, H-5), 8.43–7.34 (m, 12H, Ar-H), 6.94 (brs, 2H, NH_2_, exchangeable by D_2_O), 5.05 (s, 1H, 4H-pyran), 3.89 (q, 2H, CH_2_; *J* = 7.0 Hz), 2.27 (s, 3H, Me), 0.84 (t, 3H CH_3_; *J* = 7.0 Hz). 

*Synthesis of 3-amino-1,5-dihydro-1-(3-(naphtho[2,1-b]furan-2-yl)-1-phenyl-1H-pyrazol-4-yl)-5-oxo-pyrano[2,3-c]chromene-2-carbonitrile* (**13**): To a solution of **4a** (0.01 mol) and 4-hydroxycoumarin (0.01mol) in ethanol (50 mL) a few drops of piperidine (0.01 mL) were added. The mixture was heated under reflux for 4h and allowed to cool. The precipitated product was collected and recrystallized from dioxane to give **13** (70% yield). IR: 3,406, 3,324, 3,294 (NH_2_), 2,196 (CN), 1,708 (CO); ^1^H-NMR: 8.74 (s, 1H, H-5), 8.38–7.41 (m, 18H, Ar-H + NH_2_), 5.06 (s, 1H, 4H-pyran).

*Synthesis of 2-[(3-naphtho[2,1-b]furan-2-yl)-1-phenyl-1H-pyrazol-4-yl]methylene-hydrazono-thiazolidin-4-one* (**14**) and *2-[(3-Naphtho[2,1-b]furan-2-yl)-1-phenyl-1H-pyrazol-4-yl]methylene-hydrazono-4-methylthizole* (**15**). A mixture of **6d** (0.01 mol), ethyl bromoacetate (0.01 mol) or chloroacetone (0.01 mol) and fused sodium acetate (0.02 mol) in ethanol (40 mL) was refluxed for 2 h. The product obtained was collected by filtration, washed with water and recrystallized from acetic acid to give **14, 15**. Compound **14**: 80% yield; IR: 3,400, 3,300 (NH), 1,706 (CO); ^1^H-NMR: 11.51 (brs, 1H, NH), 9.28 (s, 1H, CH=N), 8.78(s, 1H, H-5), 8.71–7.42 (m, 12H, Ar-H), 4.00 (s, 2H, CH_2_). Compound **15**, 80% yield; IR: 3,152 (NH), 1,614 (C=N); ^1^H-NMR: 11.81 (brs, 1H, NH; exchanged by D_2_O), 9.00 (s, 1H, CH=N), 8.51 (s, 1H, H-5), 8.41–7.39 (m, 12H, Ar-H), 6.40 (s, 1H, CH-thiazoline), 2.19 (s, 3H, Me).

*Synthesis of N-(4-acetyl-4,5-dihydro-5-(3-(naphtho[2,1-b]furan-2-yl)-1-phenyl-1H-pyrazol-4-yl)-1,3,4-thiadiazol-2-yl)acetamide* (**16**): A solution of **6d** (0.01 mol), and acetic anhydride (5 mL) was heated under refluxed for 3 h. After the reaction mixture had attained room temperature, excess acetic anhydride was decomposed by water (10 mL) and the mixture was stirred for 30 min. The separated product was filtered and recrystallized from acetic acid to give **16** (60 % yield); IR: 3,248 (NH), 1,664, 1,634 (2CO); ^1^H-NMR: 11.78 (brs, 1H, NH), 8.47 (s, 1H, H-5), 8.30–7.35 (m, 12H, Ar-H), 6.44 (s, 1H, CH-thiapyrazole) 2.31 (s, 3H, Me), 2.01 (s, 3H, Me).

### Antibacterial activity

The newly synthesized compounds were screened for their antimicrobial activities *in vitro* against two species of Gram-positive bacteria *Bacillus subtilis* (NCTC- 10400)(BS), *Staphylococcus aureus* (NCTC 7447)(SA), and three Gram-negative bacteria, *Escherichia coli* (NCTC 10410)(EC), *Pseudomonas aeruginosa* (ATCC 10415)(PA), *Candida albicans* (IMRU 3669)(CA), and one fungus, *Aspergillus niger* (ATCC 6275)(AN). The activities of these compounds were tested using the disc diffusion method [19,20]. The area of zone of inhibition was measured using neomycin (30 µg mL^-1^) as standard antibiotic (Table III). The tested compounds were dissolved in *N,N*-dimethylformamide (DMF) to give a solution of 1 mg mL^−1^. The inhibition zones were measured in millimeters at the end of an incubation period of 48 h at 28 °C. *N,N*-dimethylformamide (DMF) showed no inhibition zone. 

## Conclusions

A series of novel naphtho[2,1-*b*]furan pyrazole derivatives were prepared. The antimicrobial activity of these compounds was evaluated against various Gram-positive, Gram-negative bacteria and fungi. 2-[(3-Naphtho[2,1-*b*]furan-2-yl)-1-phenyl-1H-pyrazol-4-yl]methylenehydrazonothiazolidin-4-one (**14**) showed the highest antibacterial activity, while compound **2** showed moderate activity, and the remaining tested compounds **4a-c, 5, 6, 7a,b, 8a-c, 10, 12, 13** showed no activities against any of the test microorganisms. 

## Figures and Tables

*Reagents and Conditions*: i) PhNHNH_2_, EtOH, reflux; ii) POCl_3_, DMF, reflux; iii) XCH_2_CN, EtOH, piperidine, reflux; iv) barbaturic acid, EtOH, triethylamine, reflux; v) 2-acetylnaphtho[2,1-*b*]furan (**1**); vi) aniline or *p*-chloroaniline, EtOH, reflux; vii) hydrazine derivatives, EtOH, reflux.

*Reagents and Conditions*: i) 3-methyl-1-phenyl-2-pyrazolin-5-one, EtOH, triethylamine, reflux; ii) NH_2_NH_2_·H_2_O, EtOH, reflux; iii) AcCH_2_CO_2_Et, CH_2_Cl_2_, triethylamine, reflux; iv) 4-hydroxy-coumarin, EtOH, piperidine, reflux; v) BrCH_2_CO_2_Et, CH_3_CO_2_Na, EtOH, reflux; vi) ClCH_2_COCH_3_, CH_3_CO_2_Na, EtOH, reflux; vii) Ac_2_O, reflux, viii) CH_2_(CN)_2__._

**Table I:** Physical and analytical data of new synthesized compounds **1-16**.

Comp. No.	M.P. (T/^o^C)^a^	Molecular Formula (MW)	Analysis (%) Found/calculated		
			C	H	N
**1**	108-110^a^	C_14_H_10_O_2_ (210.23)	79.95 (79.98)	4.78 (4.79)	-
**2**	172-174^a^	C_20_H_16_N_2_O (300.35)	79.96 (79.98)	5.35 (5.37)	9.30 (9.33)
**3**	180-182^a^	C_22_H_14_N_2_O_2_ (338.36)	78.00 (78.09)	4.12 (4.17)	8.20 (8.28)
**4a**	185-187^a^	C_25_H_14_N_4_O (386.40)	77.65 (77.71)	3.60 (3.65)	14.47 (14.50)
**4b**	292-294^b^	C_25_H_16_N_4_O_2_ (404.42)	74.20 (74.25)	3.95 (3.99)	13.78 (13.85)
**4c**	265-267^a^	C_25_H_16_N_4_OS (420.49)	71.35 (71.41)	3.80 (3.84)	13.25 (13.32)
**5**	350-352^a^	C_26_H_16_N_4_O_4_ (448.43)	69.60 (69.64)	3.55 (3.60)	12.35 (12.49)
**6a**	280-282^c^	C_22_H_16_N_4_O (352.39)	74.90 (74.98)	4.50 (4.58)	15.85 (15.90)
**6b**	190-192^d^	C_29_H_24_N_4_O (444.53)	78.29 (78.36)	5.35 (5.44)	12.55 (12.60)
**6c**	220-222^d^	C_25_H_22_N_4_O_2_ (410.47)	73.10 (73.15)	5.32 (5.40)	13.60 (13.65)
**6d**	228-230^b^	C_23_H_17_N_5_OS (411.48)	67.09 (67.13)	4.12 (4.16)	16.95 (17.02)
**6e**	345-347^e^	C_25_H_17_N_5_O_2_ (419.43)	71.50 (71.59)	4.05 (4.09)	16.67 (16.70)
**7a**	174-176^d^	C_29_H_23_N_3_O (429.51)	81.00 (81.09)	5.32 (5.40)	9.70 (9.78)
**7b**	170-172^d^	C_29_H_22_ClN_3_O (463.96)	75.00 (75.07)	4.70 (4.78)	9.00 (9.06)
**8**	260-262^b^	C_36_H_22_N_2_O_3_ (530.57)	81.40 (81. 49)	4.12 (4.18)	5.25 (5.28)
**10**	218-220^c^	C_32_H_22_N_4_O_2_ (494.54)	77.70 (77.72)	4.35 (4.48)	11.30 (11.33)
**12**	208-210^a^	C_31_H_24_N_4_O_4_ (516.55)	72.00 (72.08)	4.60 (4.68)	10.80 (10.85)
**13**	260-262^b^	C_34_H_20_N_44_O_4_ (548.55)	74.40 (74.44)	3.60 (3.67)	10.18 (10.21)
**14**	271-273^c^	C_25_H_17_N_5_O_2_S (451.50)	66.45 (66.50)	3.72 (3.80)	15.45 (15.51)
**15**	220-222^c^	C_26_H_19_N_5_OS (449.53)	69.42 (69.47)	4.23 (4.26)	15.54 (15.85)
**16**	255-257^c^	C_27_H_21_N_5_O_3_S (495.55)	65.38 (65.44)	4.23 (4.27)	14.00 (14.13)

^a^ From EtOH; ^b^ from dioxane; ^c^ from acetic acid; ^d^ from benzene; ^e^ from DMF

**Table II:** Significant peaks in the EI (70 ev) spectra of compounds **1, 3, 4b,c, 5, 6a-d,7b, 8, 10, 12, 13, 16**.

Comp. No.	*m/z* (intensity/%)							
	M^+^	336	310	232	193	139	77	Other peaks
**1**	210 (32)					139(100)		168(3), 113(11), 89(15), 63(38).
**3**	338 (100)		310 (20)			139 (15)	77 (62)	234 (1), 208 (1), 167 (1)
**4b**	404 (100)			232 (1)	193 (3)	139 (12)	77 (67)	360 (25), 283 (4), 257 (3),
**4c**	420 (35)		310 (1)		193 (8)	139 (15)	77 (58)	386(100), 281(2)
**5**	448 (100)					139 (7)	77 (34)	338 (14), 290 (11), 22 4(5), 181 (3)
**6a**	352 (100)	336 (66)				139 (30)	77 (44)	168 (20)
**6b**	428 (100)	336 (60)				139 (13)	77 (64)	351 (1), 284 (15), 176 (11)
**6c**	394 (100)	336 (29)				139 (5)	77 (20)	352 (10), 309 (2), 232 (3), 180 (1)
**6d**	411 (1)	336 (100)					77 (32)	308 (2), 233 (2), 20 (1), 181 (1)
**7b**	530 (65)	336 (100)		232 (5)		139 (41)	77 (28)	258 (4), 195 (16)
**8**	447 (100)	336 (7)	310 (2)					233 (3), 207 (5), 141 (15), 76 (64)
**10**	494 (100)		310 (2)		193 (5)	139 (8)	77 (31)	466 (2), 386 (25), 233 (4)
**12**	516 (2)				193 (13)	139 (5)	77 (16)	386 (100), 323 (12), 254 (4),
**13**	548 (2)		310 (5)			139 (11)		482 (15), 414 (9), 268 (12), 215 (25), 162 (23), 71 (60)
**16**	495 (45)	336(100)		232 (3)	193(4)		77 (40)	453 (18), 394 (29), 306 (4)

**Table III:** Antibacterial and antifugal activities of select synthesized compounds.

Comp. No.	Inhibition zone diameter (mm *per* 200 mcg sample)					
	*(BS)* ACTC 10400	*(SA)* NCTC 7447	*(EC)* NCTC 10410	*(PA)* ATCC 10415	*(CA)* IMRU 3669	*(AN)* ATCC 6275
**2**	++ ve	++ ve	++ ve	++ ve	- ve	- ve
**14**	+++ ve	+++ ve	+++ ve	+++ ve	+++ ve	+++ ve
**Neomycin** (30 µg mL^–1^)	+++ ve	+++ ve	+++ ve	+++ ve	+++ ve	+++ ve

^a^ *c* = 1 mg mL^–1^ of new compounds in DMF; - ve (no inhibition zone); + ve (inhibition zone up to 8 mm); ++ ve (inhibition between 8-12 mm); +++ ve (inhibition between 12-15 mm)
